# Photoacoustic imaging features of intraocular tumors: Retinoblastoma and uveal melanoma

**DOI:** 10.1371/journal.pone.0170752

**Published:** 2017-02-23

**Authors:** Guan Xu, Yafang Xue, Zeynep Gürsel Özkurt, Naziha Slimani, Zizhong Hu, Xueding Wang, Kewen Xia, Teng Ma, Qifa Zhou, Hakan Demirci

**Affiliations:** 1 Department of Radiology, University of Michigan Medical School, Ann Arbor, Michigan, United States of America; 2 Department of Biomedical Engineering, University of Michigan, Ann Arbor, Michigan, United States of America; 3 Kellogg Eye Center, University of Michigan, Ann Arbor, Michigan, United States of America; 4 Department of Ophthalmology, First Affiliated Hospital of Nanjing Medical University, Nanjing, China; 5 School of Electronics and Information Engineering, Hebei University of Technology, Tianjin, China; 6 Department of Biomedical Engineering, NIH Ultrasonic Transducer Resource Center, University of Southern California, Los Angeles, California, United States of America; Shenzhen institutes of advanced technology, CHINA

## Abstract

The purpose of this study is to examine the capability of photoacoustic (PA) imaging (PAI) in assessing the unique molecular and architectural features in ocular tumors. A real-time PA and ultrasonography (US) parallel imaging system based on a research US platform was developed to examine retinoblastoma in mice *in vivo* and human retinoblastoma and uveal melanoma *ex vivo*. PA signals were generated by optical illumination at 720, 750, 800, 850, 900 and 950 nm delivered through a fiber optical bundle. The optical absorption spectra of the tumors were derived from the PA images. The optical absorption spectrum of each tumor was quantified by fitting to a polynomial model. The microscopic architectures of the tumors were quantified by frequency domain analysis of the PA signals. Both the optical spectral and architectural features agree with the histological findings of the tumors. The mouse and human retinoblastoma showed comparable total optical absorption spectra at a correlation of 0.95 (p<0.005). The quantitative PAI features of human retinoblastoma and uveal melanoma have shown statistically significant difference in two tailed t-tests (p<0.05). Fully compatible with the concurrent procedures, PAI could be a potential tool complementary to other diagnostic modalities for characterizing intraocular tumors.

## Introduction

Intraocular tumors have relatively low incidence but could be life-threatening if not diagnosed or treated appropriately [1, 2]. The diagnosis of intraocular tumors mainly relies on the structural information and the clinical examination provided by non-invasive imaging technologies [3, 4]. However, the current imaging techniques lack to provide any detail about the histopathologic structure or functional information. Ultrasound (US) imaging, optical coherence tomography and fluorescein angiography are most commonly used diagnostic tests. US, taking advantage of the acoustic transparency of biological tissues, provides information about the shape, dimensions, location, and intrinsic structure of intraocular tumors [5, 6]. The mono-physics nature of US measurements let evaluate only the physical parameters of the backscatters in tissues such as dimensions and tissue densities without interrogating the molecular components forming these backscatters. Fluorescein angiography visualizes the vasculature in superficial structures such as the iris, retina and choroid by introducing exogenous contrast agents [7, 8]. Optical coherence tomography [9, 10] can provide a detailed structural mapping of retinal layer but limited resolution in the deeper choroidal layer [11], affected by the strong optical scattering property of biological tissues [12]. Although these techniques are helpful in the diagnosis of most intraocular tumors, atypical cases continue to be challenging. An imaging technique that can assess the molecular components and corresponding architectures inside an intraocular tumor will be helpful, important and innovative in the differential diagnosis.

Optically induced US imaging, namely photoacoustic (PA) imaging (PAI), is a non-radioactive and non-ionizing technology combining the high sensitivity of optical imaging and decent resolution of US imaging [13–17]. With focused optical illumination [18, 19], high frequency focused US transducer [20] or a combination of both [21, 22], microscopic PAI has demonstrated functional imaging capability in ophthalmology at micron-level resolution [21, 23, 24]. However, these studies are still limited to superficial imaging due to the scattering of focused illumination and the attenuation of high frequency US signal components by biological tissue [23]. Using wide-field optical illumination and reconstruction algorithms, tomographic PAI has reached imaging depth up to 5 cm [25, 26]. Tomographic PAI using multispectral illumination could therefore provide molecular information deep inside an intraocular tumor. In addition, since identical US transducer arrays are used for US and PA imaging, the two modalities are naturally matched spatially, providing coregistered structural and functional information in real time. Following procedures similar to those in quantitative US [27, 28], frequency domain PA spectral analysis (PASA) [29–33] has demonstrated the capability of quantifying the microscopic architectures formed by individual molecular components in biological tissues.

**Fig 1:**
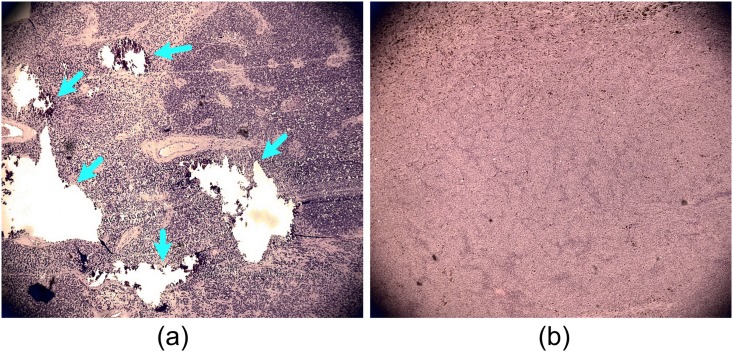
Histology photographs of retinoblastoma and uveal melanoma. (A) Retinoblastoma. (B) uveal melanoma. The arrows in (A) mark the calcification spots forming the heterogeneous tissue architecture in retinoblastoma. The slides were prepared by H&E staining. Images were taken at 4x magnification.

**Fig 2:**
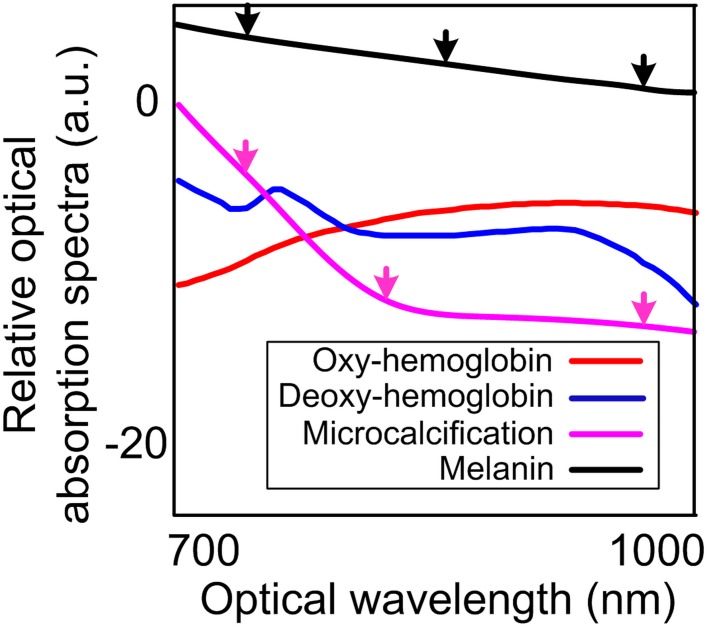
Optical absorption spectra of major molecular components in intraocular tumors [12, 34]. The black arrows indicate that the optical absorption spectrum of melanoma constantly decrease with respect to the increase of wavelengths. The optical absorption spectrum of the microcalcification shows a rapid decrease between the first two magenta arrows and a large curvature at the second magenta arrow. Both changes were captured in the PA images of retinoblastoma in the results section.

In this study, two most common primary intraocular tumors, retinoblastoma and uveal melanoma, both of which possess unique molecular and architectural features, were evaluated by our PA-US parallel imaging system [16]. Calcium component due to calcification is characteristic in retinoblastoma. Uveal melanoma originates from melanocytes that carry melanin. Histology photographs in Fig 1 show the heterogeneous architecture of retinoblastoma compared to uveal melanoma. Fig 2 shows the unique optical absorption spectra of calcium and melanin [12, 34]. These optical spectral and architectural features will be quantified in PAI for differentiating the two types of tumors.

## Methods and materials

The data acquisition and analysis approaches in our previous study [35] were used in part of this study.

### A PA-US parallel imaging system

**Fig 3:**
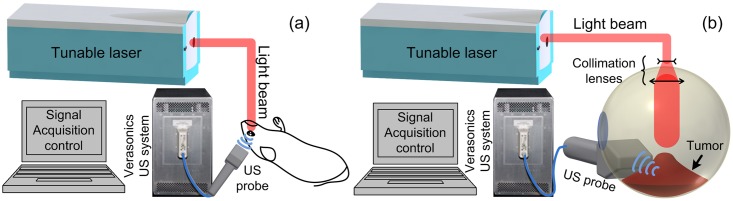
Illustration of experiment systems. (A) mouse experiment *in vivo*. (B) human eye globe imaging *ex vivo*.

Fig 3 illustrates our PA-US dual-modality imaging system [16]. The illumination of the system was provided by a wavelength tunable optical parametric oscillator (OPO) laser (Vibrant, OPOTEK, San Jose, CA) pumped by the second harmonic of a pulsed neodymium-doped aluminum garnet (Nd:YAG) laser (Powerlite, Continuum, Santa Clara, CA). The laser has a repetition rate of 10Hz, a pulse width of 8 ns and tunable ranges of 680–950 nm and 1200–2400 nm. The PA signals were acquired by a research US platform (V1, Verasonics, Redmond, WA). Illuminations at 720, 750, 800, 850, 900, and 950 nm were used. The optical absorption spectra profiles of the tumors were estimated under the consideration that optical absorption is proportional to the PA signal intensity [13]. The laser energy at the surface of the eye globes in all experiments was maintained below 20 mJ/cm^2^, which is the safety limit established by American National Standard Institute (ANSI). Considering the necessary depth for intraocular tumor imaging and the frequency range for clinical ocular US imaging, two high frequency linear US transducers were used. The human eye imaging *ex vivo* was achieved with a CL15-7 transducer array (linear array with lateral dimension of 2 cm, Philips, Andover, MA). The mouse eye imaging *in vivo* was achieved with an L22-14v transducer array (linear array with lateral dimension of 1.5cm, Verasonics, Seattle, WA). The sampling rates for the two probes were 36 MHz and 72 MHz, respectively. The time gated compensation to the signals was implemented by dividing the total imaging depth (5 cm for both animal and human tissue experiments) into 8 segments. 36 dB compensation was added to each depth segments. B-scan PAI and US images can be acquired and displayed simultaneously and continuously in real-time. Limited by the laser repetition rate, this study used a frame rate of 10 Hz. The boundaries of the tumors in the ultrasound images were depicted manually by a board certificated ocular oncologist. The tumor contours are afterwards overlaid onto the naturally coregistered PA images for quantification of the tumor optical absorption and heterogeneities.

### PAI of retinoblastoma tumors in mice *in vivo*

The laboratory animal protocol for this aim was approved by the Institutional Animal Care and Use Committee (IACUC) of University of Michigan, Ann Arbor, Michigan. 6 BLH-SV40 Tag transgenic retinoblastoma mice were examined and imaged at the 16^th^ week of age. It was confirmed that retinoblastoma tumor filled more than 50% of the globe. The visual capability of the mice was fundamentally damaged by the tumors. Both eyes of the mice were imaged, leading to 12 sample data sets. The mice were anesthetized by inhaling 1–5% vaporized isoflurane during the imaging procedures. The mouse heads were secured using surgical tapes with the eyes to be scanned facing upwards. A large volume of US coupling gel covered the eye globes. The center cross-sections of mouse eye globes were first located using US modality alone with the US transducer array placed at the upper eye lid with the eye globe exposed. The position and the orientation of the US transducer were fixed. The laser illumination was afterwards turned on and projected to the mouse eye perpendicular to the skull surface. Since the globe volume was mostly occupied by the tumor, even if the illumination hit the lens, the optical energy would be diffused instead of being focused at any spot in the eye globe and cause burning damage. Therefore, the whole eye globes were illuminated. 10% of the laser energy was split and monitored by an optical power meter (Ophir, North Logan, UT) for later signal intensity normalization. PA images were acquired and displayed in real time. The PA signals were averaged for 30 times before image reconstruction for improved signal-to-noise-ratio (SNR). The PA pixel intensities were averaged within the tumor regions and assembled with respect to the optical wavelengths, formulating the optical absorption spectrum of each tumor.

### PAI of *ex vivo* human eye globes containing retinoblastoma and uveal melanoma tumors

All procedures in this study were approved by the Institutional Review Board of the University of Michigan Medical School. Patients were diagnosed clinically and the malignancies were confirmed later histopathologically. De-identified, enucleated ocular globes containing retinoblastoma and uveal melanoma were procured at the Kellogg Eye Center in University of Michigan, and underwent imaging. Written consents were obtained before the enucleation.

The globes are imaged with the PA-US parallel imaging system immediately after enucleation. A total of 4 globes with retinoblastoma and 5 globes with uveal melanoma were imaged. For PAI imaging, the CL15-7 transducer array was used because a lower frequency array could reach deeper into the large volumes of human intraocular tumors. The eye globes were submerged in a large pool of US coupling gel. Similar to the mouse experiment, US imaging was first used to localize the tumor inside the globes. PAI was afterward turned on and both modalities acquired images in parallel. To avoid the cornea area, an 8 mm diameter fiber optics bundle was used to orient and deliver the optical illumination to the eye globe surface (Fig 3B). The cornea area was avoided as the lens could focus the optical energy onto the retina and cause visual stimulation or even damage. Optical energy scattered by the sclera could provide uniform illumination at the tumor surface and assist in deep imaging. Similar to the mouse experiments, the laser power energy was monitored by an optical power meter for signal intensity normalization. The PA signals were also averaged for 30 times before reconstruction.

Considering the large volume of human intraocular tumors, the optical absorption spectra were first solved pixel-wise within the tumor regions to explicitly show the difference between retinoblastoma and uveal melanoma. To minimize the uncertainties introduced by the slight spatial mismatch between the frames acquired at different wavelengths caused by the tiny variation of the trigger delay, the PA pixels were averaged within a sliding window of 40 axial pixels by 14 lateral pixels with a dimension of 1.7 × 1.7 mm^2^ (10 wavelengths of the 9 MHz central frequency of the US array [36]) within the tumor region. The window dimension was calculated considering the 36 MHz sampling rate and 1540 m/s speed of sound in human tissue. By combining the averaged pixel values at the same sliding window location in the PA images at all wavelengths, pixel-wise optical absorption spectra were formed. The optical absorption spectrum of each tumor was derived by averaging pixel-wise optical spectra throughout the tumor region. The optical absorption spectra of the tumors were quantified by linear regression to polynomial models. 1^st^ 2^nd^ and 3^rd^ order polynomials were attempted in search of the most appropriate model for this study:

$$S_{1}(w)=b_{1}w+a_{1}$$ (1)

$$S_{2}(w)=c_{2}w^{2}+b_{2}w+a_{2}$$ (2)

$$S_{3}(w)=d_{3}w^{3}+c_{3}w^{2}+b_{3}w+a_{3}$$ (3)

In Eqs 1–3, *S*_*i*_(*w*) is the optical absorption spectra represented by the *i*^th^ order polynomial model; *w* is the wavelengths; *a*_*i*_, *b*_*i*_, *c*_*i*_ and *d*_*i*_ are the parameters defining the models, i.e. quantitative representations of the optical spectral profiles. The parameter *a*_i_ represents the overall magnitude of the spectra. *b*_*i*_ indicates the descending rate with respect to the wavelengths. *c*_*i*_ indicates the curvature of the spectra. *d*_*i*_ indicates the change of curvature of the spectra with respect to the wavelengths. 3 sets of optical spectral parameters were derived for each tumor. The averaged parameters within the tumor regions were compared in t-tests in search for the best model for differentiating the two types of tumors. The null hypotheses of the t-test were that the parameters cannot be used to differentiate the two types of tumors. All the statistical analysis was performed using built-in functions in MATLAB (Mathworks, Natick, MA).

With the appropriate polynomial model determined, the pixel-wise optical absorption spectra were also quantified and coregistered to the US images to explicitly show the difference between the tumors.

### Texture quantification by PASA

Considering the ultrasonic resolution of PAI, the textures of the tumors in the PA images were quantified using the frequency domain power spectra of the radio-frequency (RF) PA signals. Since calcium (rich in retinoblastoma) and melanin (rich in uveal melanoma) have strong optical absorption at 720 nm (Fig 2), the PA signals at this wavelength were analyzed. The tumor area (solid magenta contour) was first selected. Similar to the previous section, the slopes were first calculated for each A-line segment of 1.7 mm (10 wavelengths correlated to the center receiving frequency of the transducer [36]) for pixel-wise analysis. We did not average the entire A-line because analyzing signal with too long temporal length will dampen the spectral information associated with the small fluctuations [36]. The full frequency bandwidth of the US transducer array, 7-15MHz, was quantitatively analyzed using PASA approach. The PASA approach was described in detail in our previous publications [29, 31, 32]. In brief, the power spectrum of an RF PA signal is fit to a linear model. The slope of the linear model represents the heterogeneity of the measured tissue. High slope value indicates more high frequency components in the PA signals and thereby more heterogeneous tissue architecture. The intercept and the midbandfit of the linear model represent the content of the molecular components with strong optical absorption at the illumination wavelength. The PASA is further illustrated in the Results section. The power spectra were first fit into linear models. The slopes and the midband-fits of the linear models were quantified. Since only tissue texture is considered, this study focused on PASA slopes. In addition to the pixel-wise analysis, the RF power spectra at all A-line segments were averaged to produce a single slope value for each tumor. The statistical difference between the slopes derived from retinoblastoma and uveal melanoma were quantified in t-test in MATLAB with the null hypothesis that the two types of tumors cannot be differentiate by their PASA slopes.

## Results

Experiment data in addition to those presented in [35] were acquired. The data were thoroughly analyzed. More solid conclusions were achieved.

### PAI of the retinoblastoma in BLH-SV40 Tag transgenic retinoblastoma mouse

**Fig 4:**
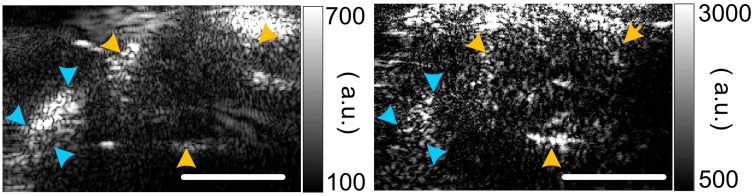
B-scan PA (acquired at 720 nm) and US images of retinoblastoma tumor in mice. The yellow arrows mark the contour of the eye globe. The cyan arrows mark the skull of the mouse. The top of the PA images show bright artifacts due to the absorption of backscattered optical energy by the US probe surface. Scale bar: 1 cm.

**Fig 5:**
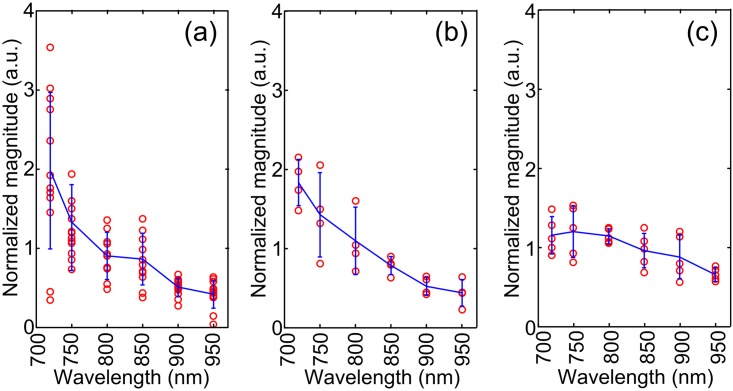
Relative optical absorption spectra complied by combining data acquired animal *in vivo* and human tissues *ex vivo*. (A) retinoblastoma in mice *in vivo*. (B) Retinoblastoma in human *ex vivo*. (C) Uveal melanoma in human *ex vivo*.

Fig 4 shows a pair of PA-US images of the mouse eye globes with retinoblastoma. The eye globes as well as the skull of the mouse were delineated in the PA images by a board certified ocular oncologist. The PA signals from the skull were due to the optical energy diffusion and the strong optical absorption of the calcium components in the skull. By compiling the averaged pixel intensity within the tumor regions from all animal subjects at all wavelengths, the characteristic optical absorption spectrum of the retinoblastoma in mouse is formulated and shown in Fig 5A.

### PAI of retinoblastoma and uveal melanoma tumors in enucleated human eyes

**Fig 6:**
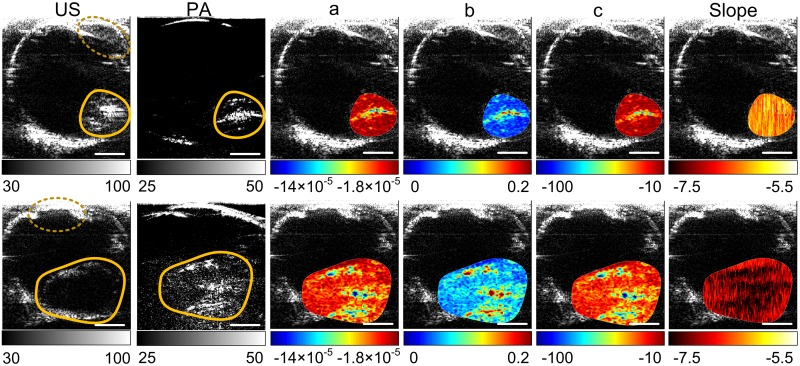
Representative US and PA images of *ex vivo* human ocular globes with tumors. Images in the upper row are from a retinoblastoma tumor. Images in the lower row are from a uveal melanoma tumor. The yellow contours mark the tumors. The pixel-wise optical spectral parameters [a, b and c in Eq 1] and PASA slopes within the tumor regions were calculated and coregistered to the US images. The units of the color axes are arbitrary. Scale bar: 1 cm.

The representative images of the retinoblastoma and uveal melanoma in *ex vivo* human eye globes were shown in Fig 6. US image shows a hyperechoic profile of retinoblastoma. Uveal melanoma appears as a hypoechoic mass in US, which does not carry much information within the tumor region. On the other hand, PA images show bright volume for both tumors.

### Characterizing the tumor types using the pixel magnitudes in PA images

**Fig 7:**
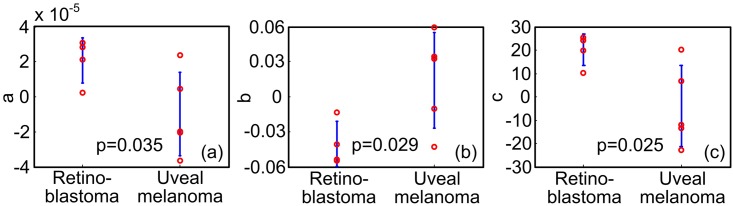
Statistical study of the quantitative features. (A)(B)(C) are the a, b and c values in Eq 2 derived from the total optical absorption spectra in Fig 5B and 5C.

A cross-correlation coefficient of 0.95 (p = 0.0032) between optical absorption spectra of the mouse and human retinoblastoma was observed. Tables 1–3 lists the quantitative parameters derived by fitting the optical absorption spectra to the polynomials in Eqs 1–3. Table 2, i.e. the regression results with Eq 2, shows the best p-values (p<0.05) in differentiating the two types of tumors. In addition, the highest order parameter in Table 3, *d*_3_ has mean and standard deviation values close to zero, indicating the higher order model is unnecessary in quantifying the absorption spectra. The statistics of the quantitative features derived using Eq 2 are shown in Fig 7. Pixel-wise optical spectral parameters were thereby calculated using Eq 2 and shown in Fig 6 columns 3–5. The higher a values of the retinoblastoma indicate that the retinoblastoma has overall high optical absorption spectral amplitudes. The lower b values of the retinoblastoma indicate that the descending rate with respect to the wavelengths of the retinoblastoma is faster than that of the uveal melanoma. The higher c value indicates that the optical absorption spectra of retinoblastoma has a larger curvature. All these quantitative features can be observed in the optical spectra shown in Fig 5B and 5C.

**Table 1:** Statistics of the optical absorption spectral parameters derived with linear model, i.e. 1^st^ order polynomial.

	Retinoblastoma		Uveal melanoma		p-values in two-tailed t-test
	Mean	Standard deviation	Mean	Standard deviation	
a_1_	6.0	2.4	2.9	1.4	0.048
b_1_	-6.0×10^−3^	2.7×10^−3^	-2.2×10^−3^	1.7×10^−3^	0.041

**Table 2:** Statistics of the optical absorption spectral parameters derived with 2^nd^ order polynomial.

	Retinoblastoma		Uveal melanoma		p-values in two-tailed t-test
	Mean	Standard deviation	Mean	Standard deviation	
a_2_	20	6.8	-3.9	17	0.025
b_2_	-0.04	0.019	0.014	0.041	0.029
c_2_	2.1×10^−5^	1.3×10^−5^	-9.8×10^−6^	2.4×10^−5^	0.035

**Table 3:** Statistics of the optical absorption spectral parameters derived with 3^rd^ order polynomial.

	Retinoblastoma		Uveal melanoma		p-values in two-tailed t-test
	Mean	Standard deviation	Mean	Standard deviation	
a_3_	38	192	-29	149	0.57
b_3_	-0.11	0.69	0.11	0.55	0.63
c_3_	9.3×10−^5^	8.2×10^−4^	1.2×10^−4^	6.8×10^−4^	0.68
d_3_	-3.1×10^−8^	3.2×10^−7^	4.4×10^−8^	2.8×10^−7^	0.72

### Shadow phenomenon in PA images and texture quantification by PASA

**Fig 8:**
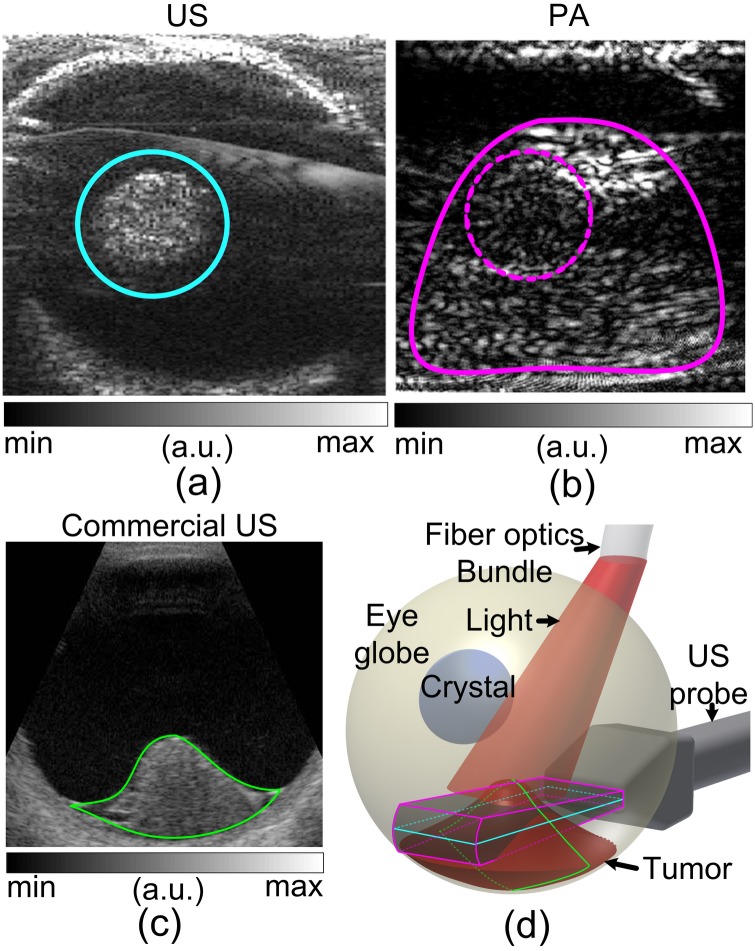
Illustration of the shadowing phenomenon in PAI. (A) Transversal cross-section of the tumor in US image. (B) PA image shows a larger tumor profile [marked by magenta contour in (D)] than that in US image [cyan contour in (C)]. (C) Sagittal cross-section of the dome-shaped uveal melanoma in US. (D) Illustration of the imaging planes in (A), (B) and (C). Since US transmission and reception were both oriented at the cyan plane in (D), only the tumor profile within the plane was shown. The larger PA tumor profile was caused by: 1) PA signals generated within the whole tumor volume due to the penetrating illumination; and 2) PA measurements using only the receiving mode of the US array, which covered the 3D volume marked by the magenta contour in (D). The optical attenuation by the apex of the tumor cast a shadow at the tumor location, marked by the dashed circle in (B).

As shown in Fig 8A and 8B, when the imaging planes were parallel to the bottom of a dome-shaped tumor (Fig 8C), the PA and US images could show different tumor profiles. This phenomenon is illustrated in Fig 8D in detail. US signals were constrained within the plane of transducer array due to the narrowly oriented ultrasound wave transmission. However, PA signals were generated throughout the tumor volume due to the diffusion of optical energy. This results in larger tumor profile in PA (solid magenta line in Fig 8B) than in US (solid cyan line in Fig 8A) images. Additionally, the tumor apex cast a shadow in the tumor due to optical attenuation (dashed magenta line in Fig 8B).

**Fig 9:**
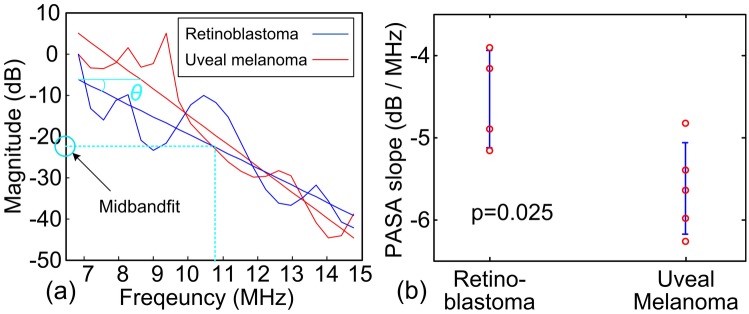
PASA of the human intraocular tumors. (A) Representative averaged PA signals spectra at 720 nm derived from the tumor samples. The solid lines are the linear fits to the signal power spectra within the probe frequency bandwidth (7-15MHz). Frequency range of 6.8–14.8 MHz was actually observed due to the limited sampling resolution. The PASA slope is tan(*θ*). Midband-fit is the magnitude of the linear model at the center of the observed frequency range (10.8 MHz for this case). (B) Statistics of the PASA slopes of retinoblastomas and uveal melanomas. The slopes derived from uveal melanomas have an average of -4.5 and standard deviation of 0.56. The slopes derived from retinoblastomas have an average of -5.6 and standard deviation of 0.60. Two-tailed t-test between the two groups has shown a p-value of 0.025.

The shadowing phenomenon in PAI could introduce uncertainty to the pixel intensity based tumor characterization. Quantifying tumor textures in the PA images could provide the alternative and reliable differential diagnosis. The tumor areas [for instance the area marked by solid magenta contour in Fig 8D] were assessed in frequency domain. Representative RF PA signal power spectra of retinoblastoma and uveal melanoma are shown in Fig 9A. More high frequency components were found in the signals from the retinoblastoma. The slope and midband-fit in PASA approach are illustrated in Fig 9A. Fig 9B shows the statistics of the PASA slopes derived from the *ex vivo* samples. The pixel-wise slopes are shown in Fig 6 last column. Significant difference between retinoblastoma and uveal melanoma was found. These findings agree with the expectation that retinoblastoma produce PA signals with more high frequency components, i.e. higher slopes, due to their more heterogeneous tissue architecture compared to uveal melanoma.

## Discussion

PAI is a hybrid modality that combines the sensitivity and contrast of optical imaging with the depth and resolution of US. US and PA image shows complementary information by using different contrast mechanisms. In US, the imaging of eye and intraocular tumor represents ultrasonic backscattering due to acoustic impedance mismatch at the interfaces of tissue structures. Retinoblastoma has heterogeneous histopathological architectures due to necrosis and calcification which are shown in US images as hyperechoic areas. Uveal melanoma has relatively homogeneous histopathological architecture with rich melanin content. The uniform internal structure in melanoma leads to a hypoechoic mass in US. In PAI, both tumors show positive contrasts over the background as a result of their strong optical absorption. The spectroscopic optical absorption profiles reflect the dominant molecular components in the tumors. For instance, the optical absorption spectra of retinoblastoma descend much faster than those of uveal melanoma in the 700–950 nm range in Fig 5, which agrees with the optical absorption spectra of calcium and melanin shown in Fig 2.

The optical absorption spectra of retinoblastoma acquired from the transgenic mice *in vivo* agree with that from human samples *ex vivo*. This validated the reproducibility of PA measurements. Although PAI is highly sensitive to the oxygenation level changes [13–16] (due to the optical absorption contrast between oxy- and deoxy-hemoglobin), strong correlation (0.95, p = 0.0032) was found between the *in vivo* and *ex vivo* optical absorption spectra. In addition, a correlation of (0.90, p = 0.015) was found between the optical absorption spectra of retinoblastoma and microcalcification. Both observations were supported by the histology that calcification is widely visible in the histology in Fig 1A.

Statistically significant differences between the two types of tumors were observed in both their optical absorption spectra and PASA slopes. Although p-values of 0.025 in two-tailed t-test were observed for both tests, differentiation using PASA slopes is a more efficient approach. This is because the absorption spectra were acquired at 6 wavelengths yet the PASA slopes were derived at only 1 wavelength. Mircoscopic architecture is a diagnostic criterion for intraocular tumors currently assessed by US [5, 37]. The fluctuations in the backscattering US signals were quantified as a representation of the heterogeneity of the tumors texture formulated by all molecular components [32]. Taking advantage of the unique optical absorption profiles of the molecular components, PASA possesses the capability of selectively appraising textures formulated by each component. Such supplemental diagnostic information could assist in better understanding of the disease pathology and improved therapeutic planning.

The optical absorption and RF spectral information were used separately in this study because we only investigated a simple differentiation between two sample groups. We will measure more samples with varied types of intraocular tumors and use more parameters for multivariate analysis in the future studies as we did in our other studies [32].

Sufficient optical energy is needed to acquire diagnostic information within the tumor located inside the globe, mostly in the posterior part. In this study, the optical energy level was kept below 20mJ/cm^2^, which is the safety limit for the skin. However, as the lens in the cornea could focus the optical energy at the retina and cause damage, this energy level is much higher than the allowable collimated laser energy density illuminating through the cornea. Diffused illumination at an energy level not interfering with normal function of the eye is crucial. In this study, of the illumination was carefully orientated by a fiber optics bundle and delivered through sclera to avoid the damage. The illumination could also be delivered through the eyelid, which could diffuse the optical energy to a safe level for the retina. We are currently investigating the illumination safety issue using rabbit eyes *in vivo* using ERG and histology. The results will be reported in a separate manuscript. On the other hand, more sensitive US probes could also reduce the required energy level for imaging the full volumes of the tumors.

## Conclusion

In summary, this is the first study that shows the feasibility of assessing intraocular tumors with PAI. A PA-US parallel imaging system and data processing techniques were developed to reveal the unique tissue optical absorption spectra and microscopic architectures of retinoblastoma and uveal melanoma. Such imaging modality may play an important role in differential diagnosis of intraocular tumors.
